# Optimization of Energy-Consuming Pathways towards Rapid Growth in HPV-Transformed Cells

**DOI:** 10.1371/journal.pone.0000628

**Published:** 2007-07-11

**Authors:** Sarit Mizrachy-Schwartz, Nataly Kravchenko-Balasha, Hannah Ben-Bassat, Shoshana Klein, Alexander Levitzki

**Affiliations:** 1 Unit of Cellular Signaling, Department of Biological Chemistry, The Alexander Silberman Institute of Life Sciences, The Hebrew University of Jerusalem, Jerusalem, Israel; 2 The Laboratory of Experimental Surgery, Hadassah University Hospital, Jerusalem, Israel; 3 The Israel National Skin Bank, Hadassah University Hospital, Jerusalem, Israel; Ordway Research Institute, Inc., United States of America

## Abstract

Cancer is a complex, multi-step process characterized by misregulated signal transduction and altered metabolism. Cancer cells divide faster than normal cells and their growth rates have been reported to correlate with increased metabolic flux during cell transformation. Here we report on progressive changes in essential elements of the biochemical network, in an *in vitro* model of transformation, consisting of primary human keratinocytes, human keratinocytes immortalized by human papillomavirus 16 (HPV16) and passaged repeatedly *in vitro*, and the extensively-passaged cells subsequently treated with the carcinogen benzo[a]pyrene. We monitored changes in cell growth, cell size and energy metabolism. The more transformed cells were smaller and divided faster, but the cellular energy flux was unchanged. During cell transformation the protein synthesis network contracted, as shown by the reduction in key cap-dependent translation factors. Moreover, there was a progressive shift towards internal ribosome entry site (IRES)-dependent translation. The switch from cap to IRES-dependent translation correlated with progressive activation of c-Src, an activator of AMP-activated protein kinase (AMPK), which controls energy-consuming processes, including protein translation. As cellular protein synthesis is a major energy-consuming process, we propose that the reduction in cell size and protein amount provide energy required for cell survival and proliferation. The cap to IRES-dependent switch seems to be part of a gradual optimization of energy-consuming mechanisms that redirects cellular processes to enhance cell growth, in the course of transformation.

## Introduction

The evolution of cancer is a complex, multi-step process characterized by an altered array of signal transduction networks and metabolic pathways [1]. Cancer cells divide faster than normal cells and their enhanced growth rate is generally believed to correlate with increased glucose flux during cell transformation [2], [3]. However, cancer cells have about the same energy available as healthy cells [2]. In order to understand better the metabolic requirements during the course of cell transformation, we have utilized a model system, which encompasses the continuum from the normal through the transformed state, based on the natural process of infection of keratinocytes by human papillomavirus (HPV). Infection with high risk HPV can lead to immortalization, but it takes decades until infected patients develop tumors. During this period the infected cells accumulate mutations and chromosomal aberrations. Risk factors like smoking are believed to affect the progression of the infected cells to the tumor stage [4]. Hence, our model system consists of primary keratinocytes (K) and keratinocytes immortalized by HPV16 [5]. To generate transformed cells, the HPV-immortalized line was extensively passaged *in vitro.* Our analysis includes cells from early passage (E), from late passage (L) and from late passage cells that underwent treatment with benzo[a]pyrene (a carcinogen that is present in cigarette smoke) to generate BP cells, which form colonies in soft agar. In validation of this model, we have shown a striking convergence of gene expression between our transformed cells and the data of Santin et al [6], who investigated changes in expression in cervical carcinoma (Kravchenko-Balasha et. al., submitted). Our model enables us to perform molecular analysis of HPV16-transformed keratinocytes at various stages, which represent steps in the transformation process, in order to try to build a global picture of the evolution of cellular transformation.

Here we report that transformed cells were smaller and divided faster than their untransformed and early-transformed counterparts, but the cellular energy flux was unchanged during transformation. Moreover, during cell transformation the protein synthesis network contracted and there was a progressive shift towards internal ribosome entry site (IRES)-dependent translation. We hypothesize that the reduction in protein synthesis allows the cell to redirect energy needed for cell survival and replication.

## Results

### Analysis of growth and energetic parameters during cell transformation

**Figure 1:**
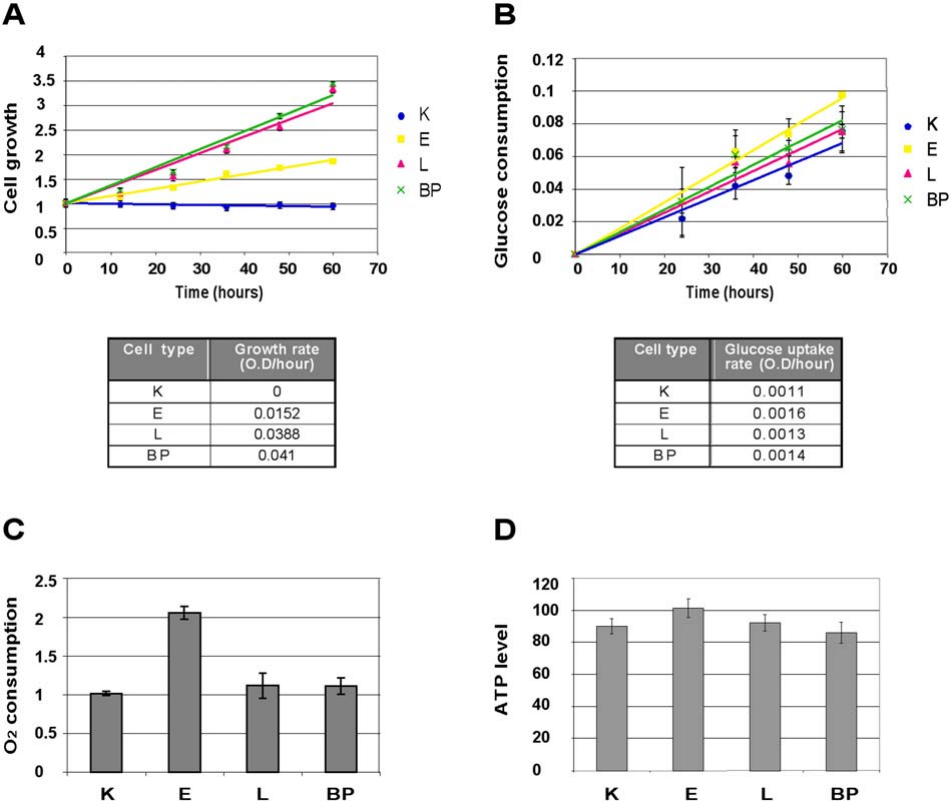
Analysis of growth and energetic parameters during cell transformation (A) Cells were grown for several hours under standard growth conditions. Growth was monitored with methylene blue staining (O.D. 630 nm, Materials and Methods). The slope of each curve represents the growth rate of the particular cell type. Error bars represent the SD of quadruplicate samples. (B) Time course of glucose uptake by K, E, L and BP cells, measured using the Amplex® Red Glucose/Glucose Oxidase Assay Kit [Molecular Probes (Invitrogen)]. Glucose consumption (O.D. 560 nm) was normalized to cell mass (O.D. 630 nm), as measured by methylene blue. The slopes represent glycolysis rates. Error bars represent the SD of duplicate samples. (C) A Clark-type oxygen electrode was used to measure oxygen consumption in primary keratinocytes (K = 1) and papilloma-transformed cells. Oxygen consumption indicates respiration rate, and was expressed as nmoles of oxygen consumed per minute per total protein amount. Error bars represent the SD of three independent experiments. (D) Cellular ATP levels were measured by the CELLTITER-GLO™ Luminescent cell viability assay (Promega). Luminescence was normalized to cell mass (O.D. 630 nm), as measured using methylene blue. Error bars represent the SD of quadruplicate samples.

Under standard growth conditions, L and BP cells divided much faster than K cells and twice as fast as E cells (Figure 1A). Interestingly however, glucose uptake (Figure 1B), remained essentially unchanged over the course of progress from K through benzo[a]pyrene induced transformation. Oxygen consumption was twice as high in E cells as in K cells, and dropped back in L and BP cells to the rate in K cells (Figure 1C). Accordingly, there was a slight increase in the steady state ATP level in E cells, whereas in K, L and BP cells the steady state ATP levels were similar (Figure 1D). Thus, E cells showed an increase in energy flux, as shown by their enhanced respiration rate. However, the energy flux in L and BP cells was similar to that in K cells, although L and BP cells had much higher rates of cell division.

### Protein synthesis in primary and transformed keratinocyte

**Figure 2:**
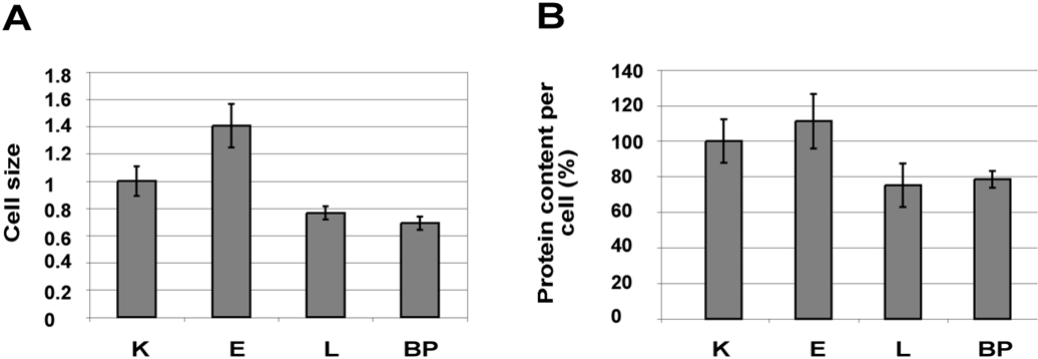
Cell size and protein amount (A) Cell size was measured using FACS. Cell size (cell diameter) as a function of FSC is presented (K = 1). Error bars represent the SD of quadruplicate samples. (B) 64 aliquots of each cell type were counted, using a counting chamber (hemocytometer). The amount of protein per 250,000 cells was measured (K = 100%), using the bound Coumassie blue method (Materials and Methods). Error bars represent the SD of quadruplicate samples.

An increase in energy production/utilization was detected in E cells, but not in L and BP cells. Moreover, light microscopy showed that E cells were clearly larger than K cells, but L and BP cells were significantly smaller than K cells. To quantify these differences, we measured cell size with a fluorescence-activated cell sorter (FACS); the forward-angle light scatter (FSC) parameter correlates with cell diameter [7]. On average, E cells were ∼30% larger in diameter than K cells (Figure 2A). Hence the increment in cell size was consistent with the increase in respiration rate per number of cells. However, L and BP cells were ∼25% smaller in diameter than K cells (Figure 2A). In other words, L and BP cells possessed about half the mass of K cells. Likewise, we measured 20% less protein per cell in L or BP cells than in K cells, and 40% less than in E cells (Figure 2B).

**Figure 3:**
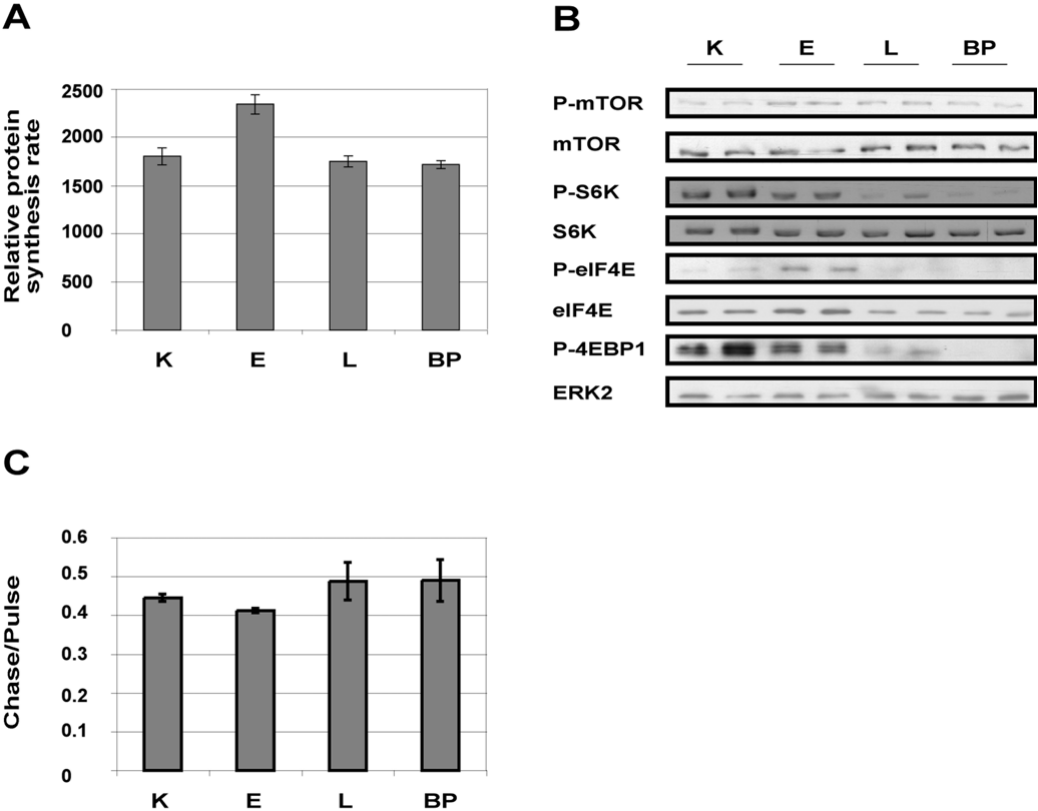
Protein synthesis and degradation in primary and transformed keratinocytes (A) Cells were seeded in 6-well plates. After 48 h, the medium was replaced with medium containing Promix (^35^S-Methionine/Cysteine), and cells were incubated for another 1 h. Labeled proteins were precipitated with trichloro acetic acid (TCA) and counted in a scintillation counter. ^35^S incorporation (cpm) was normalized to protein amount (µg), measured using the bound Coumassie blue method. Error bars represent the SD of quadruplicate samples. (B) Activity of translation factors required for cap-dependent protein synthesis. Representative western blot. Protein levels and phosphorylation of translation factors were lower in L and BP cells than in K and E cells, under standard growth conditions. ERK2 served as a gel loading control. (C) Cells were seeded in 6-well plates. After 48 h, the medium was replaced with medium containing Promix (^35^S-Methionine/Cysteine) for 1 h, washed three times, and incubated in normal growth medium for another hour. Labeled proteins were precipitated with TCA and counted in a scintillation counter. ^35^S incorporation retained during the “chase” was normalized to ^35^S incorporation during the “pulse”. Error bars represent the SD of quadruplicate samples.

Since no increase in energy production/utilization could be detected in L and BP cells in spite of their enhanced growth rate, we sought the energy source for the enhanced growth rate exhibited by L and BP cells. As protein synthesis is a major energy consuming process, which is tightly coordinated with cellular energy status [8], we examined the rate of protein synthesis in our system. We compared the protein synthesis rates of short lived proteins in K, E, L and BP cells, by pulse-labeling proteins with ^35^S-labeled methionine/cysteine. The rate of ^35^S-incorporation into protein was higher in E than in K cells, but dropped back in L and BP cells to that of K cells (Figure 3A). Consistent with this finding, the amounts and activation levels of key cap-dependent translation factors were higher in E than K cells, but lower in L and BP cells, as shown by western analysis. These factors included eukaryotic initiation factor 4E (eIF4E), (OMIM accession number 133440), phospho-eIF4E, phospho-eIF4E binding protein1 (4EBP1), (OMIM accession number 602223), phospho-mammalian target of rapamycin (mTOR), (OMIM accession number 601231), and phospho-S6 kinase1 (Figure 3B, Table 1). Since L and BP cells divide faster than K cells, but synthesize new proteins at the same rate as K cells, we infer that L and BP cells synthesize less short-lived proteins *per cell cycle* than do K cells. This is consistent with their smaller cell size and lower levels of cap-dependent translation factors. Moreover, using pulse chase analysis we saw no increase in protein degradation during cell transformation (Figure 3C). Hence the decrease in protein amount in L and BP was not due to enhanced degradation, but rather to decreased protein synthesis. The shrunken repertoire of proteins synthesized by L and BP cells should represent a significant energy saving to these cells.

**Table 1:** Short description of the functions of key factors involved in cap-mediated translation.

Protein name	Function
eIF4E	A rate-limiting translation factor, plays an important role in mRNA translation by binding the 5′-cap structure of the mRNA and facilitating the recruitment to the mRNA of other translation factors and the 40S ribosomal subunit [27].
4EBP1	Inhibits 5′-cap-dependent mRNA translation by binding and inactivating eIF4E. Phosphorylation of 4EBP1 releases eIF4E, allowing initiation of translation [27].
mTOR	The mTOR pathway regulates protein synthesis by directly phosphorylating S6K and 4EBP1. mTOR activation depends on several inputs including nutrients (amino acid) and energy (ATP) [28], [29].
S6 kinase1	Activated (phosphorylated) S6 kinase1 promotes protein synthesis and is an important regulator of cell size [28].

### IRES-dependent translation increases in transformed cells

**Figure 4:**
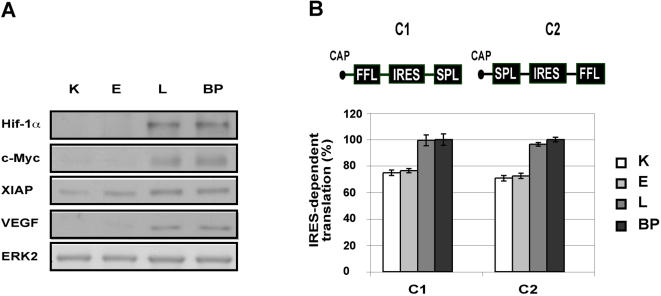
IRES-dependent translation increases in transformed cells (A) Levels of proteins that are subject to both cap and IRES-mediated translation under standard growth conditions. A representative western blot is shown. ERK2 served as a gel loading control. (B) Reciprocal constructs for cap-dependent and IRES-dependent translation of firefly luciferase (FFL) and renilla luciferase (SPL) constructs are illustrated. The ratio of IRES-dependent to cap-dependent product is represented by SPL/FFL activity for construct 1 (C1, left) and by FFL/SPL activity for construct 2 (C2, right). Bars represent the SD of duplicate samples

These findings led us to propose that the reductions in cell size and amount of protein provide energy required for functions that are augmented during cellular transformation, such as cell replication. We further hypothesized that during the course of cellular transformation, the cells progressively economize on the repertoire of proteins synthesized, in favor of proteins directly involved in cell proliferation and cell survival. Indeed, analysis of proteins known to be involved in cellular transformation and survival showed marked increases in the amounts of c-Myc (OMIM accession number 190080), X-linked inhibitor of apoptosis (XIAP) (OMIM accession number 300079), hypoxia-inducible factor 1α (Hif-1α), (OMIM accession number 603348) and vascular endothelial growth factor (VEGF), (OMIM accession number 192240) in L and BP cells (Figure 4A), confirming that the expression of certain proteins was induced, despite the overall decrease in cap-dependent translation.

**Figure 5:**
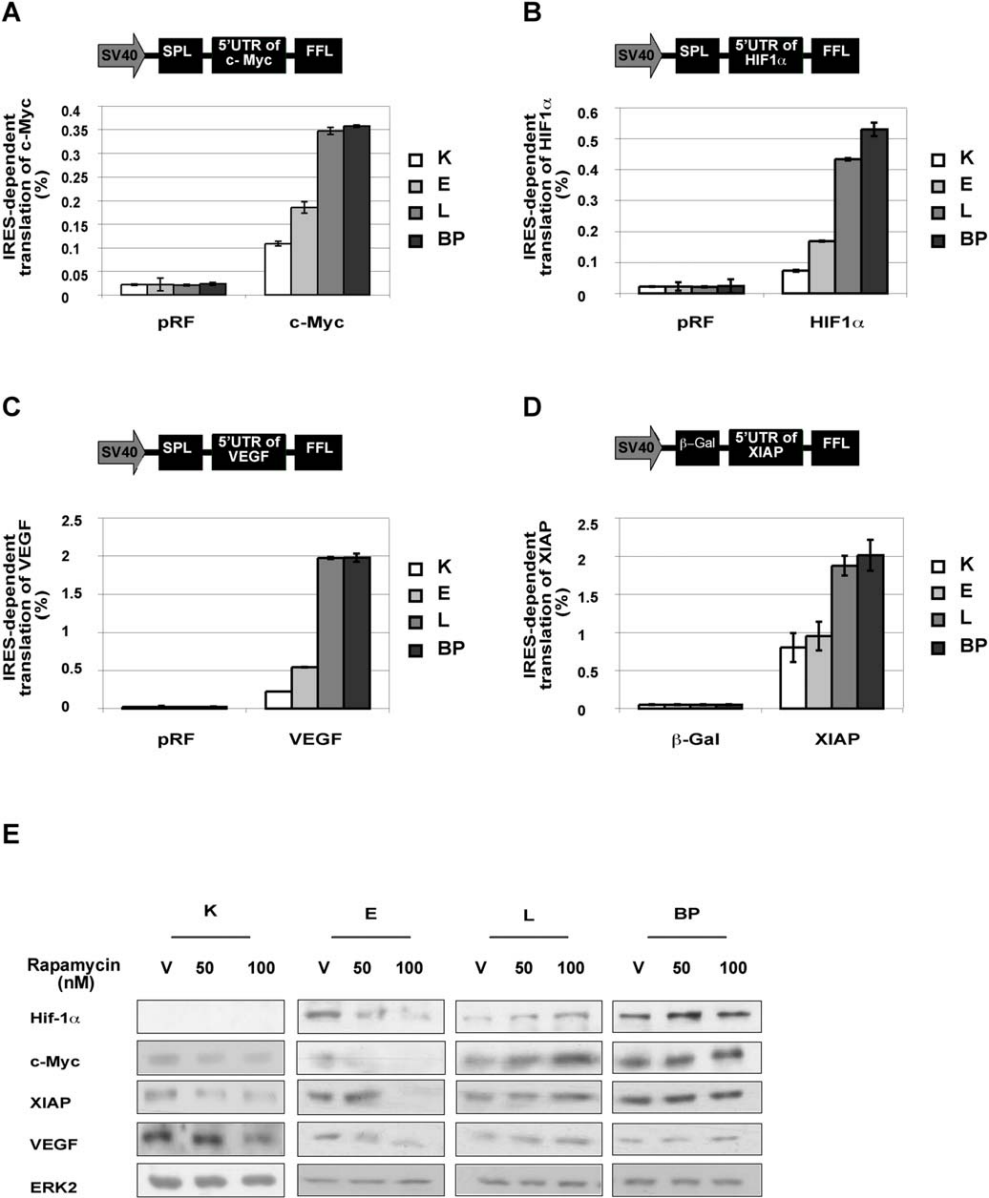
IRES vs. cap-dependent translation. Bicistronic constructs incorporating the 5′UTRs of c-Myc, HIF-1α, VEGF or XIAP were transfected into the various cell types. The ratios of IRES/cap activities were analyzed under standard growth conditions for the various cells. (A, B, C) For c-Myc, HIF-1α and VEGF, the IRES/cap ratios were represented by FFL/SPL activity (pRF = vector control). (D) For XIAP, the IRES/cap ratio was represented by chloramphenicol acetyl transferase (CAT)/β-galactosidase activity (β-gal = vector control). Error bars represent the SD of duplicate samples. (E) Differential effects of rapamycin treatment on translation of proteins subject to both cap and IRES-translation, in K, E, L, and BP cells. ERK2 served as a gel loading control. Representative western analysis is shown. (V = vehicle)

Although cap-mediated protein synthesis is the dominant mechanism for generating proteins, the proteins we demonstrated to be highly expressed in L and BP cells – namely, c-Myc, XIAP, Hif-1α and VEGF – can be translated by both cap and IRES-dependent mechanisms [9]–[12]. In view of the increased levels of the dually translatable proteins and of the known association between decreased eIF4E levels (see Figure 3B) and IRES-dependent translation [13], we assayed IRES-mediated translation in the transformed cells. Using a luciferase reporter system that utilizes reciprocal bicistronic plasmids from which both cap and IRES-dependent translation can be measured [14], we showed that IRES-dependent translation is enhanced relative to cap-dependent translation in L and BP cells (Figure 4B). This finding was corroborated by the demonstration that IRES-dependent translation from the 5′-untranslated regions(UTRs) of c-Myc, HIF1α, VEGF and XIAP was much more extensive in L and BP cells than in K cells (Figure 5A–D). Furthermore, rapamycin, a selective inhibitor of mTOR, and hence of cap-related translation, blocked the accumulation of c-Myc, XIAP, VEGF, and Hif-1α in K and E cells, but not in L and BP cells (Figure 5E). Thus, although cap-dependent translation was reduced in L and BP cells, IRES-dependent translation was enhanced.

### Regulation of protein synthesis during cell transformation

**Figure 6:**
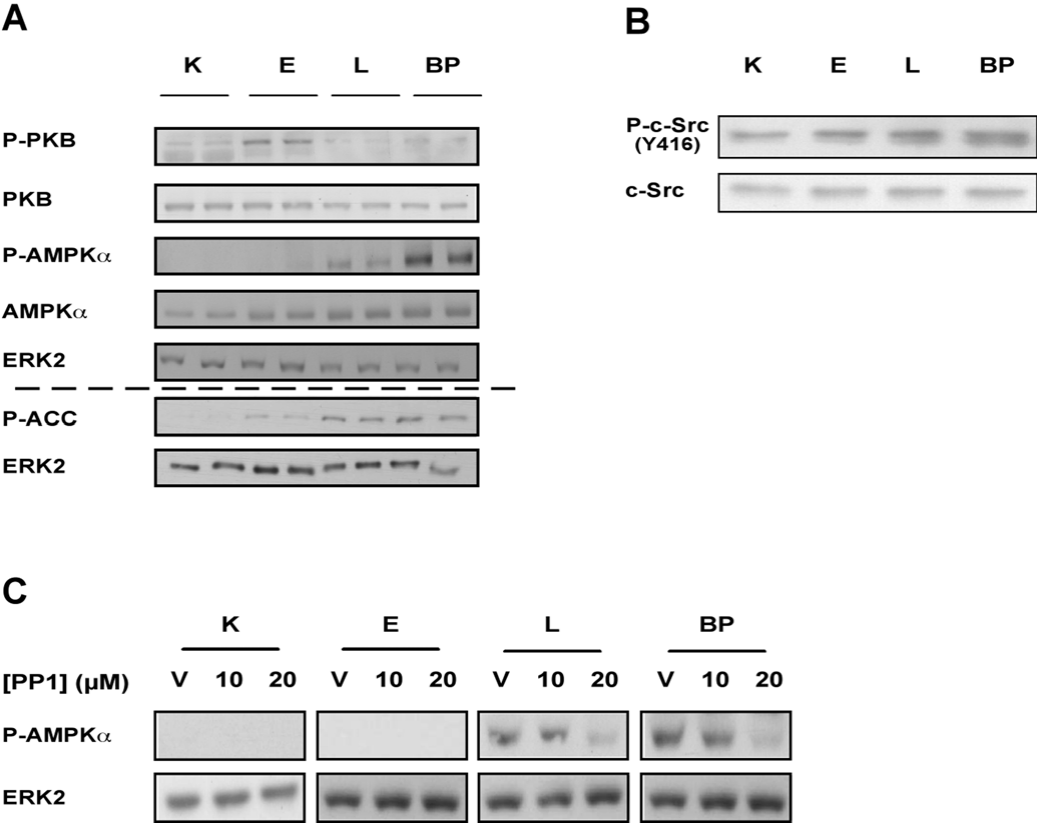
Regulation of protein synthesis during cell transformation (A) Representative western analysis of K, E, L and BP cell lysates, using the indicated antibodies. ERK2 served as a gel loading control. PKB and AMPK were visualized on one blot and P-ACC on a separate blot. The activity of PKB dropped and the activity of AMPK rose in L and BP. (B) The phosphorylation of c-Src (Y416) was induced during transformation. The amount of total c-Src protein (phosphorylated and non-phosphorylated) remained unchanged. (C) Cells were treated with PP1 to inhibit c-Src. After 4 h cells were lysed, and western blot analysis was performed. ERK2 served as a gel loading control. Representative western analysis is shown.

In order to understand in more detail the mechanism that regulates the switch from cap to IRES-dependent translation, we examined the activities of proteins involved in the regulation of protein synthesis. Protein kinase B (PKB), (OMIM accession number 164730) is known to regulate intracellular ATP levels and is also a negative regulator of AMP-activated protein kinase (AMPK) (OMIM accession number 602739), which inhibits mTOR through the activation of Tuberous sclerosis 2 (TSC2), (OMIM accession number 191092) [15]. Western blot analysis revealed that the level of the activated (phosphorylated) form of PKB was lower in L and BP cells than in K and E cells, whereas the activity of AMPKα was higher, as measured by its state of phosphorylation, and by the phosphorylation status of the AMPK target acetyl-CoA carboxylase (ACC) (OMIM accession number 200350) (Figure 6A).

Recently published data demonstrate that AMPK can be induced not only by elevation of the AMP/ATP ratio, but also by c-Src (OMIM accession number 190090), independently of the AMP/ATP ratio in the cell [16], [17]. Since AMPK was activated although ATP levels remained unchanged during progressive transformation from K to BP, we examined whether c-Src regulates AMPK activity in the more transformed cells. During the course of K to BP transformation, c-Src was activated (Figure 6B). Furthermore, in the presence of the c-Src inhibitor, PP1 [18], the level of phosphorylated AMPK decreased in L and BP cells (Figure 6C). These data support the proposition that AMPK is activated in L and BP cells by c-Src, whilst the ATP levels of the cells remain unchanged.

**Figure 7:**
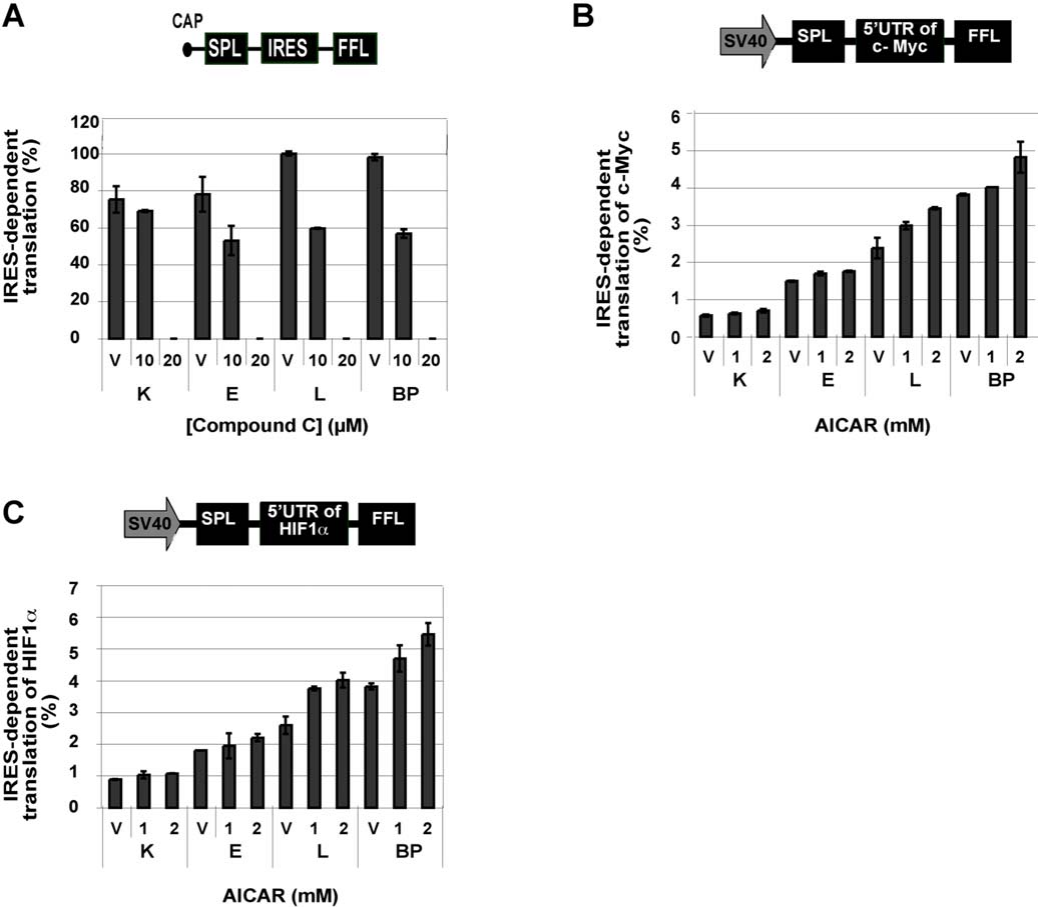
AMPK as a regulator of protein synthesis (A) Cells were transfected with the reciprocal bicistronic constructs and IRES vs. cap-dependent translation was measured in the presence of the AMPK inhibitor (compound C). IRES-dependent translation was reduced in all the cell types when AMPK was inhibited. Similar results were obtained with the reciprocal construct (not shown). Error bars represent the SD of duplicate samples. (B, C) Cells were transfected with the c-Myc or HIF-1α bicistronic constructs. Activation of AMPK led to an increase in FFL/SPL (IRES/cap) activity. Error bars represent the SD of duplicate samples. (V = vehicle)

We next examined more directly the possible involvement of AMPK in the regulation of protein synthesis. Addition of the AMPK inhibitor, compound C [19], led to a decline in the ratio of IRES to cap-dependent translation (Figure 7A). Correspondingly, activation of AMPK by 5-aminoimidazole-4-carboxamide-1-β-D-ribofuranoside (AICAR) [15], led to an increase in the IRES fraction of particular mRNAs known to be subject to both IRES and cap-dependent translation. For example, IRES-dependent translation from the c-Myc and Hif-1α 5′UTRs increased under AICAR treatment (Figure 7B,C). Thus, the increase in the IRES-mediated translation fraction in the transformed cells appears to be a consequence of increased c-Src and AMPK activities.

**Figure 8:**
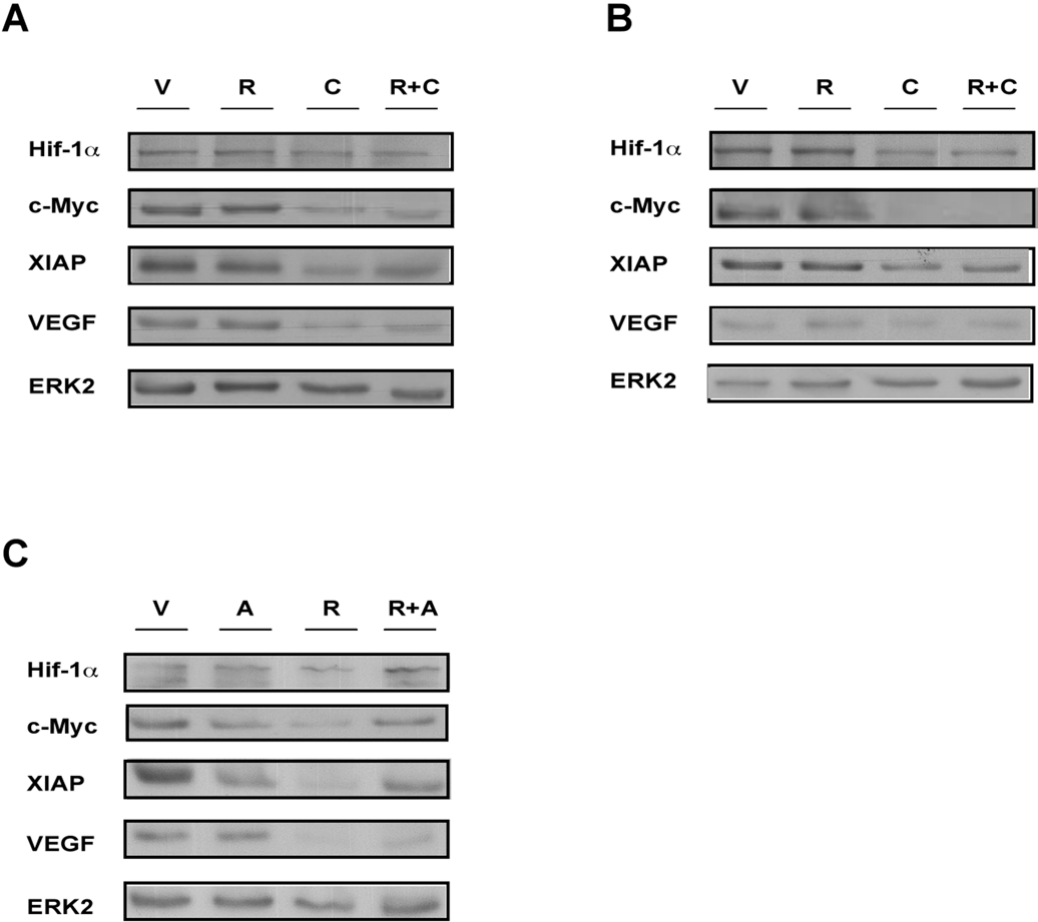
AMPK is involved in the regulation of IRES-dependent translation. L (A) and BP cells (B) were treated with 150 nM rapamycin (R) or 10 µM compound C (C) or 150 nM rapamycin in the presence of 10 µM compound C (R+C) for 24 hours. (C) E cells were treated with 150 nM rapamycin (R) or 2 mM AICAR (A) or 150 nM rapamycin in the presence of 2 mM AICAR (R+A) for 24 hours. Levels of proteins subject to both cap and IRES-translation were analyzed by Western blot using the indicated antibodies. ERK2 served as a gel loading control.

AMPK has been documented to be a negative regulator of cap-dependent translation [15]. In order to examine whether AMPK is also a direct regulator of IRES-dependent translation, we treated L and BP cells with compound C. Inhibition of AMPK with compound C caused a reduction in the levels of c-Myc, XIAP, VEGF and Hif-1α in L and BP cells. However, inhibition of mTOR by rapamycin had no effect (Figure 8A,B). Thus, in L and BP cells, the levels of c-Myc, XIAP, VEGF and Hif-1α proteins are dependent upon AMPK activity, implying that AMPK might be involved directly in the regulation of IRES-dependent translation. Next, we inhibited the mTOR pathway in K and E cells. We had previously shown that this pathway is less active in L and BP cells than in K and E cells (Figure 3B). As described above, rapamycin treatment alone reduced protein levels of c-Myc, XIAP, VEGF, and Hif-1α (Figure 8C). On the other hand, activation of AMPK with AICAR in K (data not shown), and E cells (Figure 8C), had no effect on the levels of these proteins. These results confirm that in K and E cells, the levels of these proteins are dependent on mTOR.

Finally, we examined whether activation of AMPK is involved in the switch from cap to IRES-dependent translation. Activation of AMPK by AICAR in the presence of rapamycin led to increased levels of c-Myc, XIAP, VEGF, and Hif-1α proteins in E cells (Figure 8C), but not in K cells (data not shown). Thus, the inhibition of the mTOR pathway and the coordinate activation of AMPK in immortalized E cells was able to mimic at least partially the evolution that L and BP cells underwent during the course of transformation.

## Discussion

In order to follow the biochemical changes that occur when cells are transformed, we established an in-vitro cell system where we have a continuum of cells, from normal human keratinocytes, through consecutive passages of HPV16 immortalized cells, all the way to extensively passaged, benzo[a]pyrene treated cells that form colonies in soft agar. This system enables us to follow the transition from normal cells to transformed cells. The relevance of this model to naturally occurring tumorigenesis is strongly supported by the remarkable similarity between the changes in gene expression pattern of the HPV16 transformed keratinocytes, and the changes in gene expression when cervical carcinoma was compared to uninfected cervix [6], where the degree of similarity is highest between BP cells and cervical carcinoma (Kravchenko-Balasha et al, submitted).

We observed an increase in cellular energy flux in the early stage of transformation as compared to normal keratinocytes (Figure 1C). This increase is consistent with the increases in growth rate, cell size, and protein amount per cell (Figure 1A, Figure 2A,B). The E stage is tetraploid, but devoid of gross chromosomal translocations (data not shown). At later stages of cell transformation L and BP cells showed similar rates of energy production/utilization to the normal K cells (Figure 1B-D). Yet, the more transformed L and BP cells proliferate much more rapidly than K and E cells (Figure 1A). L and BP cells are smaller, have lower protein content (Figure 2A,B), synthesize less protein and show a severe reduction in cap-dependent translation and in the levels of the protein factors executing this type of translation (Figure 3A,B). Since L and BP cells divide faster than K cells, but have similar rates of protein synthesis, we inferred, and then experimentally verified, that there is a net reduction in protein amount per cell in L and BP as compared to K cells, which is indeed reflected in the reduced cell size.

We note that our focus was on short-lived proteins. Long-lived proteins constitute the majority of proteins in the cell. However, short-lived proteins are key components in the cell, involved in signal transduction, cell-cycle control, transcription, apoptosis and many other fundamental processes. Due to their short half lives, they are more frequently translated. Hence, this group of proteins comprises a large fraction of the proteins synthesized per cell cycle.

We hypothesized that the energy conserved by the shrinkage of the protein synthesis network is channeled to functions, such as cell replication, which are enhanced during the course of cellular transformation. Indeed we show that the levels of proteins known to be involved in cellular transformation increase (Figure 4A). Since we observed a reduction in cap-dependent translation factors, but an increase in specific proteins that can be translated by both cap and IRES-dependent mechanisms [9]–[12], we investigated the role of IRES-dependent translation in the transformed cells. IRES-dependent translation was first identified in picornaviruses, but can also be used for cellular mRNAs of genes involved in the control of cellular proliferation, survival, and death [20]. It is likely that IRES-mediated translation consumes less energy than cap-mediated translation. During the initiation of IRES-dependent translation the ribosome binds at or close to the first AUG and scanning does not occur, so the initiation of IRES-dependent translation does not consume ATP [21],[22].

A connection between IRES-mediated translation and cancer has been tacitly assumed [20], but has never been demonstrated. Here we show that there is a direct connection between cellular transformation and IRES-dependent translation. We reveal that the reduction in cap-dependent translation is accompanied by a global switch to IRES-dependent translation during cellular transformation (Figure 4B). Furthermore, we show that the expression of several cancer-related genes becomes contingent upon IRES-mediated translation as the cells become transformed (Figure 5).

It has been suggested that IRES-dependent translation is enhanced under various cellular stresses, such as hypoxia, amino acid starvation, serum starvation and low dose irradiation. Under these conditions global protein synthesis is inhibited [23]. In our experiments, no extraneous stress was imposed. L and BP cells, however, might suffer from intrinsic stress. For instance, L and BP cells show extensive aneuploidy and chromosomal translocations (data not shown), and such aberrations have been associated with cellular stress [24]. Moreover, we have found that L and BP cells have a low stress response threshold and are hyper-sensitive to DNA damage, as expected for transformed cells (Kravchenko-Balasha et al., submitted). In a parallel study, we have shown that the transcriptional repertoire is severely reduced in L and BP cells (Kravchenko-Balasha et al., submitted). These global contractions in cellular networks presumably reduce cellular robustness and contribute to hyper-sensitivity to stress, but may also provide the additional energy required for rapid proliferation.

What triggers the switch to IRES-mediated translation? The increase in the fraction of IRES-dependent translation was dependent upon AMPK activity (Figure 7 and Figure 8), and the increased levels of AMPK in the transformed cells were dependent on the elevated c-Src activity in these cells (Figure 6B,C), rather than on the AMP:ATP ratio in the cells. Regulation of AMPK by c-Src has been described in other cell systems and seems to be a bona-fide signaling module [16], [17]. We conclude that activation of AMPK by c-Src signals the cell to save energy by reducing ATP-consuming processes such as cap-mediated protein synthesis.

Activation of AMPK has been reported to decrease cap-dependent translation [15]. The increase in the proportion of IRES-translated protein could result from a reduction in cap-dependent translation. However, here we show that inhibition of AMPK, but not of mTOR, leads to a reduction in the levels of c-Myc, XIAP, VEGF and Hif-1α proteins in L and BP cells. Furthermore, in E cells, AMPK activation in the presence of rapamycin, led to increased levels of these proteins (Figure 8). Thus, AMPK may act both to decrease cap-mediated translation and to increase IRES-mediated translation. In K cells, no increase in the levels of the described proteins was observed during combined treatment with AICAR and rapamycin (data not shown). However, K cells have less AMPK to begin with (Figure 6A). Furthermore, it is important to note that IRES dependent translation may also be regulated by additional factors that are not present in K cells.

Transformed cells communicate less with their neighbors and seem to adopt a more “primitive” way of life. The shift to IRES-mediated translation, which resembles the translational machinery of bacteria [25], [26], may be another manifestation of the dedifferentiation of transformed cells.

In summary, we have shown that E cells divide faster than K cells, have an enhanced protein synthesis rate and are larger in size. The energy needed for these functions may derive from their enhanced respiration rate (Figure 1C). However, L and BP cells divide even faster than E cells, but their cellular energy flux is like that of K cells. We speculate that the enhanced growth rate of the transformed cells evolved at the expense of other ATP consuming processes, not essential for cellular growth. Our results imply a correlation between the reduction in energy consuming cap-dependent translation, AMPK activation and IRES-dependent translation. We postulate that the switch from cap to IRES-dependent translation during progressive HPV16-induced cell transformation, contributes to the overall re-channeling of cellular energy towards pathways dedicated to enhanced cell growth.

## Materials and Methods

### Cell culture

Primary keratinocytes and HPV16-immortalized cells were maintained in Keratinocyte Growth Medium (KGM): DMEM, 25% Nutrient mixture F-12 (HAM), 10% fetal bovine serum, 5 µg/ml insulin, 0.4 µg/ml hydrocortisone, 10^−10^ M cholera toxin, 10 ng/ml epidermal growth factor, 1.8×10^−4^ M adenine, 5 µg/ml transferrin, 2×10^−9^ M T3, 100,000 U/L penicillin, 100 µg/L streptomycin, 0.1 mg/ml amphotericin. Primary keratinocytes were cultured from small biopsy specimens. The HPV16-immortalized keratinocytes had been transfected with the genome of the human papillomavirus, HPV16 [5]. Early passage (passage 20, E) represents cells that underwent about 50 doublings after transfection. Late passage (passage 270, L) represents cells that underwent about 1000 doublings. BP cells were derived from L, by benzo[a]pyrene treatment. L cells were treated with benzo[a]pyrene (5 µM) for 30 days to establish a clone able to form colonies in soft agar.

### Western blot analysis

Primary and transformed keratinocytes were plated at the concentration indicated in each assay. Where indicated, medium was replaced with medium containing PP1 (SYNTHOSE Ltd), rapamycin (ICN Biochemical Inc), AICAR (Toronto Research Chemicals Inc) or compound C (Merck), 48 h after initial plating, and cells were further incubated for the indicated length of time. Cells were then washed with PBS (50 mM NaH_2_PO, 50 mM Na_2_HPO_4_, 0.77 M NaCl) and lysed in sample buffer (40% glycerol, 0.2 M Tris pH 6.8, 20% β-Mercaptoethanol, 12% Sodium dodecyl sulfate (SDS), Bromo Phenol Blue). Samples were boiled for 5 min and equal amounts of protein (as determined using bound Coumassie blue [below]) from each sample were separated by SDS-PAGE. Proteins were transferred to a nitrocellulose membrane. Membranes were blocked with 5% low fat milk in TBST (10 mM Tris-HCl, pH 7.4 (TBS), 0.2% Tween 20, 170 mM NaCl). Afterwards, membranes were reacted with the relevant antibodies. Blots were then reacted with a peroxidase-coupled secondary antibody (Jackson Immunoresearch Inc.), and visualized by ECL. When needed, blots were stripped of antibodies by incubating in 2% SDS, 10 mM β-mercaptoethanol, 62.5 mM Tris-HCl pH 6.8 at 55 °C for 20 min, washed with TBST, and then blocked with 5% milk in TBST and re-probed. The following antibodies were used: Anti-phospho-ACC-S79, anti-PKB, anti-phospho-PKB-T308, anti-mTOR, anti-phospho-mTOR-S2448, anti-phospho-4E-BP-T70, anti-eIF4E, anti-phospho-eIF4E-S209, anti-S6 kinase1, anti-phospho-S6 kinase1-T389, anti-AMPK, anti-phospho-AMPKα-T172, anti-phospho-c-Src-Y416 from Cell Signaling Technology; anti-Erk2, anti-c-Myc, anti-VEGF from Santa Cruz Biotechnology Inc.; anti-XIAP, anti-Hif-1α from BD Transduction Laboratories; anti-c-Src from hybridoma mab 327. Each experiment was repeated at least 3 times.

### Cell transfection and drug treatment

K cells (5×10^5^/well), E cells (1×10^5^/well), L and BP cells (8×10^4^/well) were seeded onto 6-well plates (Falcon). Cells were transfected 48 h later with 1 µg DNA/well, using Lipofectamine and Lipofectamine Plus reagents (Invitrogen) according to the manufacturer's protocol. 24 h post-transfection, AMPK activator, AICAR (Toronto Research Chemicals Inc) or AMPK inhibitor, commonly known as compound C (Merck) was added as indicated and cells were incubated for an additional 24 h. The cells were then lysed.

### Reporter assays

Cells were transfected with the appropriate reporter plasmids, as above.

#### Luciferase assay

36 h after transfection, cells were harvested and protein extracts were prepared by lysis with Reporter Lysis Buffer (Promega). FFL and SPL activities were assayed with a dual-luciferase kit (Promega) using a Luminoskan Ascent (Thermo Labsystem luminometer). For the bicistronic constructs containing the 5′UTR of c-Myc, HIF-1α and VEGF, FFL activity was normalized to SPL activity, in order to correct for transfection efficiencies. Each experiment was repeated at least 3 times.

#### CAT assay

After incubation for 36 h, a CAT assay was performed using the CAT ELISA kit (Roche), according to the manufacturer's protocol. CAT activity was normalized to β-galactosidase activity in order to correct for transfection efficiencies. The experiment was repeated 3 times.

The bicistronic translation reporter plasmids, pEF-FFL-IRES-SPL and pEF-SPL-IRES-FFL [14], were kindly provided by R. Fukunaga (see Figure 4B). pRF and the bicistronic plasmid containing the IRES element of c-Myc [9] were kindly provided by A. E. Willis (see Figure 5A). The bicistronic plasmids containing the IRES element of VEGF and Hif-1α [12] were kindly provided by G. J. Goodall (see Figure 5B,C). The β-gal/CAT bicistronic construct and the βgal/CAT bicistronic construct containing the IRES element of XIAP [10] were kindly provided by S. Baird (see Figure 5D).

### Methylene blue cell quantification assay

Cells were fixed on 6-well or 96-well plates using 2.5% gluteraldehyde, washed three times with DDW and left to dry overnight. Plates were stained with methylene blue solution (1% methylene blue in 0.1 M borate buffer pH 8.5) at room temperature for 60 minutes. Plates were repeatedly washed with DDW and left to dry overnight. Color was extracted with 0.1 M HCl by shaking at 37°C for 60 minutes. Samples were loaded onto 96-well ELISA plates and O.D. was measured at 630 nm. The experiment was repeated at least 3 times.

### Protein synthesis rate determination

Cells were plated in 6-well plates, and after 48 h the medium was replaced with medium containing 100 µCi/ml Promix (^35^S-Methionine/Cysteine), (Amersham Biosciences). After 1 h cells were washed three times with PBS, and lysed in sample buffer. Labeled proteins were precipitated with TCA and counted in a scintillation counter. ^35^S incorporation was normalized to protein amount, measured using the bound Coumassie blue method (below). The experiment was repeated at least 3 times.

### Pulse chase analysis

48 h after plating, cells were pulsed for 1 h with medium containing 100 µCi/ml Promix. Cells were then washed three times with PBS, samples were withdrawn and lysed at this point. The remaining cells were incubated in KGM, chased for another hour and lysed. Labeled proteins were precipitated with TCA and counted in a scintillation counter. ^35^S retained during the “chase” was normalized to ^35^S incorporation in ”pulse”. The experiment was repeated 3 times.

### Glucose uptake assay

Cells were grown as described. A 2 µl sample of medium was taken every 12 h for the duration of 60 h, and was diluted 250-fold. The assay was performed using the Amplex Red/Glucose Oxidase kit (Molecular Probes). Absorbance was measured at 560nm and normalized to the cell mass as measured with methylene blue. The experiment was repeated 3 times.

### ATP measurement

K (2×10^4^/well), E (10^4^/well), L and BP cells (8×10^3^/well) were seeded in 96 mm dishes. The CELLTITER-GLO™ Luminescent cell viability assay (Promega) was performed according to the manufacturer's protocol. Basal ATP steady state levels were measured 36 h after plating. Each cell type was tested at least 3 times.

### O_2_ consumption

K, E, L, and BP cells, cultured in 6-well plates as described above, were trypsinized in exponential phase. Aliquots of 5×10^6^ cells were resuspended in 50 ml of KGM and incubated at 37°C. 180 min later cells were resuspended again in 1.6 ml of KGM and the rate of O_2_ consumption was recorded in a polarographic cell at 37°C using a Clark oxygen electrode. Cell respiration was expressed as the rate of decrease of oxygen concentration. Cell oxygen consumption was calculated by analyzing the slope of the line on the chart recorder and was expressed as nanomoles of oxygen consumed per minute per cell mass. The experiment was repeated 3 times.

### Cell size analysis

Cells were pelleted, washed once with PBS, and resuspended in 500 µl PBS. For measurement of cell size using FSC, samples were analyzed using a FACS (FACSCalibur; Becton Dickinson). The experiment was repeated 3 times.

### Protein determination using bound Coumassie blue stain

Samples (5 µl) and BSA standards (1–10 µg) were spotted onto filter paper strips (0.5×1.5 cm, Whatman 3 mm). Filter papers were stained with Coomassie brilliant blue G-250 (Bio-Rad, USA, 2.5 g/L in 40% methanol, 10% acetic acid) and unbound dye was washed with destain solution (20% methanol, 7% acetic acid). Bound Coomassie stain was extracted from the filter papers with 3% SDS, and the absorbance of the solution was measured at 590 nm. Protein amounts were determined using a bovine serum albumin calibration curve.

### Determination of protein amount per cell

Using a cell counting chamber placed under a phase contrast optical microscope, 64 samples from each cell type were counted. Then the amount of protein in 250,000 cells using the bound Coumassie blue method, were determined. The experiment was repeated 2 times.
